# Quadriceps miR-542-3p and -5p are elevated in COPD and reduce function by inhibiting ribosomal and protein synthesis

**DOI:** 10.1152/japplphysiol.00882.2018

**Published:** 2019-01-24

**Authors:** Roser Farre-Garros, Jen Y. Lee, S. Amanda Natanek, Martin Connolly, Avan A. Sayer, Harnish Patel, Cyrus Cooper, Michael I. Polkey, Paul R. Kemp

**Affiliations:** ^1^Molecular Medicine Section, National Heart and Lung Institute, Imperial College, South Kensington Campus, London, United Kingdom; ^2^National Institute for Health Research Respiratory Biomedical Research Unit at Royal Brompton and Harefield National Health Service Foundation Trust and Imperial College, London, United Kingdom; ^3^Medical Research Council Lifecourse Epidemiology Unit, University of Southampton, Southampton General Hospital, Southampton, United Kingdom; ^4^Academic Geriatric Medicine, Faculty of Medicine, University of Southampton, Southampton, United Kingdom; ^5^Ageing Geriatrics and Epidemiology Research Group, Institute of Neuroscience, Faculty of Medical Sciences, Newcastle University, Newcastle upon Tyne, United Kingdom; ^6^National Institute for Health Research Newcastle Biomedical Research Centre, Newcastle upon Tyne Hospitals National Health Service Foundation Trust and Newcastle University, Newcastle upon Tyne, United Kingdom

**Keywords:** chronic obstructive pulmonary disease, microRNA, mitochondrial and cytoplasmic ribosomes

## Abstract

Reduced physical performance reduces quality of life in patients with chronic obstructive pulmonary disease (COPD). Impaired physical performance is, in part, a consequence of reduced muscle mass and function, which is accompanied by mitochondrial dysfunction. We recently showed that miR-542-3p and miR-542-5p were elevated in a small cohort of COPD patients and more markedly in critical care patients. In mice, these microRNAs (miRNAs) promoted mitochondrial dysfunction suggesting that they would affect physical performance in patients with COPD, but we did not explore the association of these miRNAs with disease severity or physical performance further. We therefore quantified miR-542-3p/5p and mitochondrial rRNA expression in RNA extracted from quadriceps muscle of patients with COPD and determined their association with physical performance. As miR-542-3p inhibits ribosomal protein synthesis its ability to inhibit protein synthesis was also determined in vitro. Both miR-542-3p expression and -5p expression were elevated in patients with COPD (5-fold *P* < 0.001) and the degree of elevation associated with impaired lung function (transfer capacity of the lung for CO in % and forced expiratory volume in 1 s in %) and physical performance (6-min walk distance in %). In COPD patients, the ratio of 12S rRNA to 16S rRNA was suppressed suggesting mitochondrial ribosomal stress and mitochondrial dysfunction and miR-542-3p/5p expression was inversely associated with mitochondrial gene expression and positively associated with p53 activity. miR-542-3p suppressed RPS23 expression and maximal protein synthesis in vitro. Our data show that miR-542-3p and -5p expression is elevated in COPD patients and may suppress physical performance at least in part by inhibiting mitochondrial and cytoplasmic ribosome synthesis and suppressing protein synthesis.

**NEW & NOTEWORTHY** miR-542-3p and -5p are elevated in the quadriceps muscle of patients with chronic obstructive pulmonary disease (COPD) in proportion to the severity of their lung disease. These microRNAs inhibit mitochondrial and cytoplasmic protein synthesis suggesting that they contribute to impaired exercise performance in COPD.

## INTRODUCTION

A reduction in muscle mass and strength occurs both in response to chronic disease and as part of normal human aging (9), and loss of muscle function reduces quality of life by limiting ability to perform normal daily tasks and is associated with increased mortality (7, 36). One chronic disease for which skeletal muscle wasting is a common comorbidity is chronic obstructive pulmonary disease (COPD) (19), and similarly, in this condition, wasting and reduced muscle function are associated with poorer quality of life and increased mortality (8, 35).

Muscle function is affected both by the amount of muscle, which predominantly affects strength (12), and its oxidative capacity, which predominantly affects endurance. Muscle oxidative capacity is dependent on the number and function of mitochondria in the tissue (31). In COPD patients, there is a reduction in both muscle mass (33) and mitochondrial oxidative capacity (13, 20). The loss of muscle mass arises from an imbalance in protein turnover with a relative increase in protein breakdown compared with protein synthesis, and diverse studies have reported changes in components of both degradative and synthetic pathways, but, taken together, these data do not convey a consistent conclusion suggesting that there are multiple factors that regulate mass (15).

There are also a number of studies examining changes in mitochondria in the muscle of COPD patients. Together they show both reduced mitochondrial density and mitochondrial dysfunction. The factors contributing to reduced mitochondrial density include reduced mitochondriogenesis with lower levels of proliferator-activated receptor-γ coactivator-1α and mitochondrial transcription factor A. The molecular mechanisms leading to mitochondrial dysfunction are less clear. Reduced activity of electron transfer complexes that contain mitochondrially encoded proteins has been reported (37) as have changes in the respiratory control ratio (29). Consistent with these observations, a recent transcriptomic analysis of COPD muscle showed marked reduction in the expression of mitochondrial genes with weighted gene correlation network analysis identifying a mitochondrially enriched gene module as a major factor associated with pulmonary function and exercise capacity (41). Changes were also seen in mitochondrial dysfunction associated with aging and in patients with intensive care unit-acquired weakness (ICUAW) (3, 14, 37).

One mechanism by which this change in the activity of mitochondrial complexes that contain mitochondrially encoded proteins may occur is through the inhibition of mitochondrial translation. We recently showed that the microRNA (miRNA) miR-542-3p inhibited the expression of mitochondrial ribosomal proteins, reduced mitochondrial rRNA in particular the 12S rRNA leading to a reduction in the 12S:16S mitochondrial rRNA ratio, and reduced mitochondrial membrane potential (11). This miRNA also targets cytoplasmic ribosomal proteins suggesting that it would reduce maximal protein synthesis so may contribute to a loss of muscle mass. Elevation of this miRNA was observed in a small cohort of patients with severe COPD, but the association of this miRNA with muscle function in COPD patients was not analyzed further in our prior report (11). We also did not quantify the expression of mitochondrial RNAs to determine whether there was evidence for mitochondrial ribosomal stress in COPD patients.

The current study therefore was designed to investigate the expression of miR-542-3p/5p in the quadriceps of a second, larger cohort of COPD patients, compare it to physical performance, and quantify the expression of mitochondrial rRNAs. The expression of these miRNAs was determined in the muscle of healthy older individuals to identify any association with muscle dysfunction in the absence of overt disease. We also determined the effect of miR-542-3p on cytoplasmic protein synthesis.

## METHODS

### Subjects

All subjects gave informed consent before inclusion in the relevant study, which was approved by the appropriate ethical review bodies as detailed for each cohort. All procedures were carried out in accordance with the Helsinki Declaration.

#### COPD cohort.

COPD subjects (*n* = 52) and controls (*n* = 16) were part of a larger study described by Natanek et al. (23). The patients had COPD according to the Global Initiative in Obstructive Lung Disease (GOLD) guidelines 2004 (30) and were enrolled from clinics at the Royal Brompton Hospital. Patients with a diagnosis of heart, renal, or liver failure, a systemic inflammatory or metabolic disorder, or a moderate/severe exacerbation (i.e., requiring antibiotics, oral steroids, or hospitalization) in the preceding 4 wk were excluded. Healthy age-matched controls (16 in total) were recruited by advertisement. All subjects gave written informed consent and the protocol was approved by the Royal Brompton and Harefield National Health Service Trust Research Ethics Committee (Studies 06/Q0404/35 and 06/Q0410/54). Measurements of lung volume, using plethysmography; carbon monoxide transfer factor, using the single breath technique (CompactLab, Jaeger, Germany); and postbronchodilator spirometry were performed according to American Thoracic Society/European Respiratory Society guidelines (40). Blood gas tensions were measured in arterialized capillary earlobe blood. Bioelectrical impedance was used to determine fat-free mass index (FFMI; Bodystat 1500, Bodystat, UK) as described previously (34).

Quadriceps strength was determined by measuring supine isometric maximal voluntary contraction (MVC) as described previously (35) and physical performance measured as 6-min walk distance, 5 min after bronchodilator treatment [American Thoracic Society 2002 guidelines (1)]. Vastus lateralis muscle biopsies were obtained under local anesthesia by percutaneous needle biopsy of the in the midthigh using the Bergstrom technique (2).

#### Hertfordshire Sarcopenia Study cohort.

The study protocol was approved by the Hertfordshire Research Ethics Committee (study 07/Q0204/68) and all participants gave written informed consent. Muscle mass and strength together with gait speed and a timed-up-and go (TUG) test were ascertained; Hertfordshire Sarcopenia Study (HSS) methods have been previously described (25). Muscle biopsy was performed by percutaneous conchotome biopsy of the vastus lateralis under local anesthesia (24).

### RNA Analysis

#### Quantification of rRNA.

RNA was extracted using Trizol, and transcripts were reverse transcribed into cDNA using random primers using the Quantitect RT Kit (Qiagen) and quantified by real-time qRT-PCR as described previously using the primers described in Table 1 (10). rRNA expression was normalized to hypoxanthine phosphoribosyltransferase (HPRT) expression (human samples) or to the geometric mean of HPRT and β2-microglobin (cell culture samples) in the same sample using the ΔΔCT method.

**Table 1.** Primers used in this study

Target	Forward	Reverse
12S	CCCAAACTGGGATTAGATACCC	GTTTGCTGAAGATGGCGGTA
16S	GCCTGTTTACCAAAAACATCAC	CTCCATAGGGTCTTCTCGTCTT
HPRT	GCTATAAATTCTTTGCTGACCTGCTG	AATTACTTTTATGTCCCCTGTTGACTGG
β2-Microglobulin	TGCTGTCTCCATGTTTGATGTATCT	TCTCTGCTCCCCACCTCTAAGT

HRPT, hypoxanthine phosphoribosyltransferase.

#### Quantification of miRNAs.

miRNAs were reverse transcribed using MultiScribe Reverse Transcriptase and Megaplex RT Primers (human pool A, Version 3.0; Applied Biosystems) according to the manufacturer’s instructions. The samples were heated to 85°C for 5min to terminate the reactions then stored at −80°C. The cDNAs were preamplified using Megaplex PreAmp Primers (Applied Biosystems) for 12 cycles of 95°C for 15 s and 60°C for 4 min, and the reactions were terminated by heating to 99.9°C for 10 min. The resulting cDNA was diluted to 100 μl by addition of 0.1× TE buffer pH 8.0 (Qiagen) and stored at −80°C. Primers and probes for miR-542-3p and miR-542-5p were purchased for each test gene from Applied Biosystems, and amplification was carried out according to the manufacturer’s instructions. Each reaction was performed in duplicate, and the average Ct value was normalized to the corresponding geometric mean of U6 and RNU48 using the ΔΔCt method. RNA isolated from cells was analyzed using single RT reactions.

### Cell Culture

HLCN-M2 cells were maintained in skeletal muscle growth medium (PromoCell) supplemented with 20% FCS, as previously described (42). All experiments were performed on cells as myoblasts. RNA was extracted using Trizol; mRNA and miRNAs were quantified as described above.

#### miRNA transfection.

Six-thousand two-hundred and fifty cells were seeded into each well of a 96-well plate. After 24 h, cells were transfected with mirVana miRNA mimics with each well treated with a mixture containing 0.5 µl of 20 µM mirVana miRNA mimic(s) and 0.5 µl Lipofectamine 2000 (ThermoFisher) prepared according to the manufacturer’s instructions.

#### RNA extraction.

Forty-eight hours after microRNA transfection, RNA was extracted from LHCN-M2 cells using the CellAmp Direct RNA Prep Kit (TaKaRa) according to the manufacturer’s instructions. miRNA and rRNA were analyzed as described above.

### Protein Analysis

#### Western blotting.

Western blotting for RPS23 was carried out as previously described (18) but using rabbit anti-RPS23 (Novus) at 1:100.

#### Protein synthesis.

Forty-eight hours after miRNA transfection, LHCN-M2 cells were serum and leucine-starved by incubation in leucine-free DMEM. Two hours later, the medium was replaced with leucine-containing DMEM supplemented with 130 nM of IGF-1 (Cambridge Bioscience), and the cells were incubated for 45 min before addition of 100 ng/ml puromycin (Sigma) and harvested 30 min later, and protein was extracted. Three-hundred nanograms of the extracted protein were diluted in 200 µl of 50 mM sodium bicarbonate and incubated in a 96-well plate well at 37°C for 2 h. The wells were washed (1 time in PBS) then blocked with 200 µl PBS containing 5% BSA (wt/vol; PBS-BSA) for 30 min at room temperature. The solution was replaced with 100 µl of 100 ng/ml anti-puromycin (Millipore) diluted in PBS-BSA and the samples were incubated for 1 h at room temperature. After being washed two times in PBS, 100 µl of sheep anti-mouse (GE Healthcare) was added at a 1/10,000 dilution in PBS-BSA. The samples were washed four times in PBS before 100 µl of TMB substrate (Sigma) was added, and the samples were incubated for 15 min. The reaction was stopped with 100 µl of stopping solution (Sigma), and absorbance was determined at 450 nm.

### Statistical Analysis

Gene expression data from human studies were log transformed to stabilize variance and produce a normal distribution. Gene expression data from in vitro studies were normalized to the mean value for the appropriate control data set. Statistical analysis was performed in Aabel (Gigawiz). Pearson correlations were used to identify linear correlations after visual inspection of scatter plots. Differences between groups were calculated by Student’s *t*-test for normally distributed data and by Mann-Whitney *U*-test for nonparametric data. Differences between multiple groups were identified by ANOVA with post hoc testing using a Bonferroni-Dunn correction.

### Bioinformatic Analysis

The quadriceps gene expression profiles of the majority of the patients included in this study have been published previously (*n* = 41 patients and *n* = 12 controls) (41). Correlation analysis was performed within the WGCNA R package using robust biweight midcorrelation to determine the correlation coefficients. Gene set enrichment analysis was then performed to identify groups of genes associated with the miRNAs comparing the correlation coefficients for genes that were significant at *P* < 0.02. To determine the association of the miRNAs with mRNAs for mitochondrial ribosomal proteins, the geometric mean of the target proteins individually and for all detected mitochondrial small ribosomal proteins was calculated and compared with miR-542 expression in the same sample.

## RESULTS

### Patient Demographics

By design and consistent with a diagnosis of COPD, the patients had poorer lung function than controls by all measures. The patient cohort included 11 patients with GOLD 1–2, 25 patients with GOLD 3, and 16 patients with GOLD 4 COPD. There was no difference in FFMI between patients and controls, but patients had poorer exercise performance measured both as 6-min walk distance and quadriceps MVC. The patients also had lower daily activity levels than the controls. The demographic data are presented in Table 2.

**Table 2.** Physiological characteristics of the COPD cohort

	Control (*n* = 16)	COPD (*n* = 52)
Sex (male/female)	7, 9	30, 22
Age, yr	66 ± 8	66 ± 8
Smoking history,^a^ pack-yr	0 (0, 10)	44 (30, 60)***
Weight,^a^ kg	67 (61, 74)	66 (59, 77)
BMI,^a^ kg/m^2^	24.8 (23.5, 26.2)	23.3 (21.4, 26.2)
FFMI,^a^ kg/m^2^	16.1 (15.3, 17.2)	15.3 (14.5, 16.8)
FEV_1_,^a^ %pred	107.6 (100.6, 112)	40.7 (27.2, 48.4)***
RVTLC	38 ± 6	59 ± 8***
TL_CO_,^a^ %pred	92.3 (83.0, 98.1)	45.0 (32.1, 52.4)***
6-Min walk, m	621 ± 84	387 ± 125***
6-Min walk, %pred	126 ± 12	76 ± 24***
PV̇o_2_,^a^ %pred	99 (88, 111)	46 (38, 58)***
SGRQ,^a^	2 (0, 8)	54 (45, 61)***
Quadriceps MVC, kg	34.7 ± 10.6	28.2 ± 8.9*
Quadriceps MVC, %pred	78 ± 19	63 ± 14**
Locomotion time,^a^ min/12 h	96 (84, 127)	41 (26, 56)***
Movement time, %12 h	23 ± 6	13 ± 5***

Values are means ± SE or median (interquartile range). BMI, body mass index; FFMI, fat free mass index; FEV_1_, forced expiratory volume in 1 s; RVTLC, ratio of the reserve volume to total lung capacity; TL_CO_, transfer capacity of the lung for CO; SGRQ, St George’s respiratory questionnaire; MVC, maximal voluntary contraction.

* *P* < 0.05.

** *P* < 0.01.

*** *P* < 0.001. Significance determined by Student’s *t*-test except for variables denoted ^a^, where *P* values were calculated by Mann Whitney *U*-test.

### Expression of miR-542-3p/5p in the Quadriceps of Patients with COPD

**Fig. 1.**
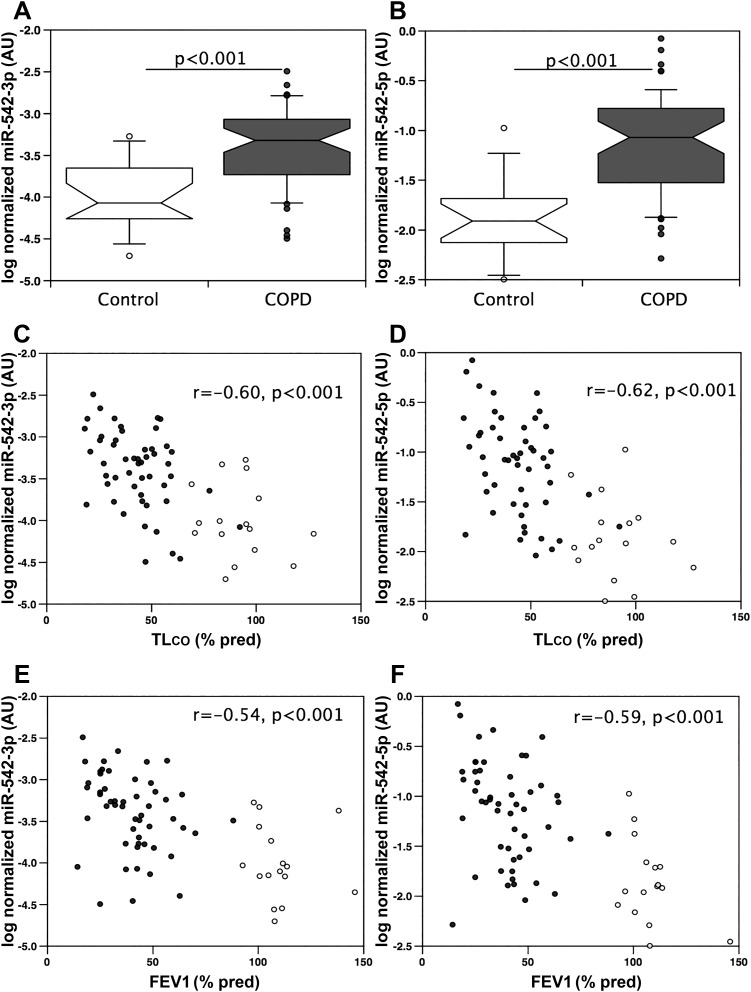
Expression of the miR-542-3p and miR542-5p is increased in the quadriceps of chronic obstructive pulmonary disease (COPD) patients and associated with disease severity. miR-542-3p and miR-542-5p were quantified in muscle biopsies from patients with COPD (*n* = 52) and controls (*n* = 16). *A* and *B*: miR-542-3p (*A*) was elevated 5.5-fold (*P* < 0.001) and miR-542-5p (*B*) was elevated 5.9-fold (*P* < 0.001). AU, arbitrary units. *C*–*F*: comparison of the expression of both microRNAs (miRNAs) with measurements of lung function transfer factor for CO percent predicted (TL_CO_%; *C* and *D*) and forced expiratory volume in 1 s in percent predicted (FEV_1_%; *E* and *F*) showed that expression of these miRNAs in the quadriceps was associated with disease severity. miRNA expression was normalized to the expression of U6 and RNU48 in the same samples. Data were analyzed by *t*-test and Pearson correlations shown are for the entire cohort. Patients are shown as closed circles and controls as empty circles.

**Fig. 2.**
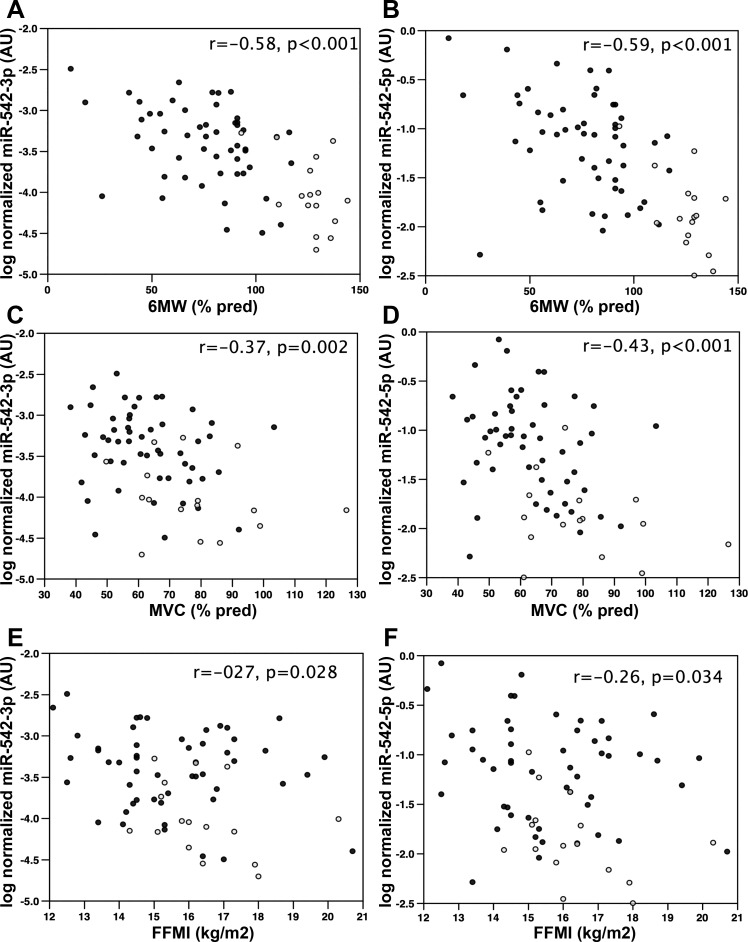
The miR-542-3p and 542-5p are associated with physical performance in chronic obstructive pulmonary disease (COPD). miR-542-3p and miR-542-5p were quantified in muscle biopsies from patients with COPD (*n* = 52) and controls (*n* = 16). *A* and *B*: comparison of the expression quadriceps of both microRNAs (miRNAs) with 6-min walk distance (6MW) %predicted showed an inverse association both in the whole cohort and in the patients as a subgroup [miR-542-3p, (*A*) and miR-542-5p (*B*)]. AU, arbitrary units. *C* and *D*: comparison of the expression of both miRNAs with strength (maximal voluntary contraction (MVC) %predicted showed a weak inverse association in the whole cohort but not in the patients as a subgroup [miR-542-3p (*C*) and miR-542-5p (*D*)]. *E* and *F*: comparison of the expression quadriceps of both miRNAs with FFMI showed a weak inverse association in the whole cohort but not in the patients as a subgroup [miR-542-3p (*E*) and miR-542-5p (*F*)]. miRNA expression was normalized to the expression of U6 and RNU48 in the same samples. Pearson correlation coefficients shown are for the entire cohort. Patients are shown as closed circles and controls as empty circles.

The expression of miR-542-3p/5p was determined in a cohort of COPD patients (both sexes and all GOLD grades) and controls. Both miRNAs were elevated in the muscle of COPD patients compared with controls (542-3p: 5.5-fold, *P* < 0.001 and 542-5p: 5.9-fold, *P* < 0.001; Fig. 1, *A* and *B*) and highly correlated with each other (*r* = 0.95, *P* < 0.001). Quadriceps expression of these miRNAs was inversely associated with lung function measured either as transfer capacity of the lung for CO percent predicted (TL_CO_%) (542-3p: *r* = −0.60, *P* < 0.001 and 542-5p: *r* = −0.62, *P* < 0.001 for the whole cohort; and 542-3p: *r* = −0.44, *P* = 0.001 and 542-5p: *r* = −0.42, *P* = 0.002, for the patients alone; Fig. 1, *C* and *D*) or as forced expiratory volume in 1 s in percent predicted (FEV_1_%) 542-3p: *r* = −0.54, *P* < 0.001 and 542-5p: *r* = −0.59, *P* < 0.001 for the whole cohort; and 542-3p: *r* = −0.38, *P* = 0.006 and 542-5p: *r* = −0.29, *P* = 0.035, for the patients only; Fig. 1, *E* and *F*). Furthermore, the miRNAs were inversely proportional to exercise performance measured as 6-min walk distance expressed, as percent predicted, in both the whole cohort (542-3p: *r* = −0.58, *P* < 0.001 and 542-5p: *r* = −0.59, *P* < 0.001; Fig. 2, *A* and *B*) and in the patients alone (542-3p: *r* = −0.037, *P* = 0.006 and 542-5p: *r* = −0.35, *P* = 0.012). Although both miRNAs were weakly inversely associated with strength in the patients and controls considered together (measured as MVC% predicted, 542-3p: *r* = −0.37, *P* = 0.002 and 542-5p: *r* = −0.43, *P* < 0.001; Fig. 2, *C* and *D*) and FFMI (542-3p: *r* = −0.27, *P* = 0.028 and 542-5p: *r* = −0.26, *P* = 0.034; Fig. 2, *E* and *F*), neither miRNA was associated with strength in the patients alone (542-3p: *r* = −0.22, *P* = 0.109 and 542-5p: *r* = −0.27, *P* = 0.054) or FFMI (542-3p: *r* = −0.177, *P* = 0.210 and 542-5p: *r* = −0.172, *P* = 0.224). The associations did not differ significantly between men and women (data not shown).

**Fig. 3.**
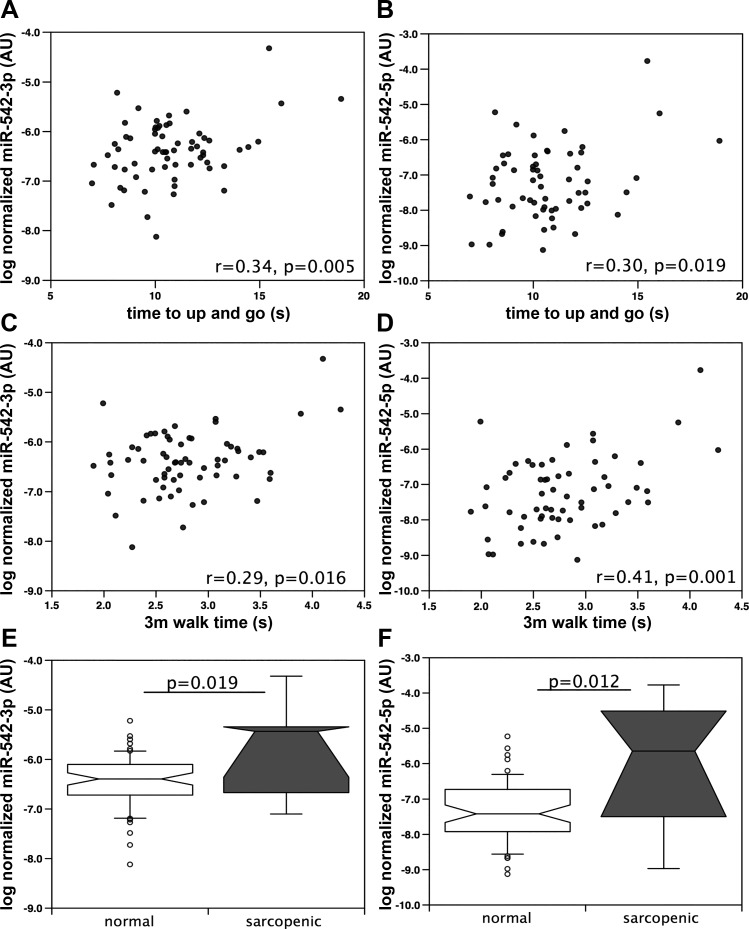
The miR-542-3p and -5p are associated with physical performance in older people. *A*–*D*: miR-542-3p and miR-542-5p were quantified in quadriceps biopsies from older individuals enrolled in the Hertfordshire Sarcopenia Study (HSS) (*n* = 64) and correlated with physical performance measured using the timed up and go test (*A* and *B*) and 3-min gait speed (*C* and *D*). AU, arbitrary units. *A*–*D*: both miR-542-3p (*A* and *C*) and miR-542-5p (*B* and *D*) were associated with poor physical performance on both tests. *E* and *F*: furthermore, in the patients identified as sarcopenic according to European Working Group on Sarcopenia in Older People guidelines (*n* = 5) both microRNAs (miRNAs) [miR-542-3p (*E*) and miR-542-5p *(F*)] were elevated compared with those who were not sarcopenic (*n* = 59). The data shown for miR-542-3p are taken from all 64 samples; however, 4 samples did not amplify for miR-542-5p (3 nonsarcopenic and 1 sarcopenic), so these data come from 56 control and 4 sarcopenic individuals. miRNA expression was normalized to the expression of U6 and RNU48 in the same samples. Pearson correlation coefficients shown and statistical difference was calculated by Student’s *t*-test.

To further determine whether the expression of these miRNAs was also associated with muscle function in healthy seniors, we also measured the expression of the miRNAs in samples from the HSS (demographics in Table 3). In normal healthy older men from the HSS, both miRNAs were associated with poorer physical performance as indicated by 3-min walk time and time to up and go 6-min TUG test (Fig. 3, *A*–*D*). Although neither miRNA was associated with FFMI in the HSS participants, both were elevated in those defined as having sarcopenia (Fig. *3, E* and *F*).

**Table 3.** Physiological characteristics of HSS cohort

	Nonsarcopenic (*n* = 59)	Sarcopenic (*n* = 5)
Weight,^a^ kg	83 (72, 92)	69 (66, 71)*
BMI,^a^ kg/m^2^	27 (25, 29)	26 (25, 27)
FFMI,^a^ kg/m^2^	18.5 (17.5, 19.3)	16.2 (15.9, 17.1)**
FEV_1_,^a^ %pred	106 (98, 117)	108 (103, 111)
TUG time, s	10.4 ± 1.7	13.7 ± 4.7**
3-Min walk time,^a^ s	2.7 ± 0.4	3.4 ± 1.0**

Values are means ± SE or median (interquartile range). HSS, Hertfordshire Sarcopenia Study (HSS); BMI, body mass index; FFMI, fat free mass index; FEV_1_, forced expiratory volume in 1 s; TUG time, time to “get up and go.”

* *P* < 0.05.

** *P* < 0.01.

Significance determined by Student’s *t*-test except for variables denoted ^a^, where *P* values were calculated by Mann-Whitney *U*-test.

### Do COPD Patients Show Evidence of Mitochondrial Ribosomal Stress?

**Fig. 4.**
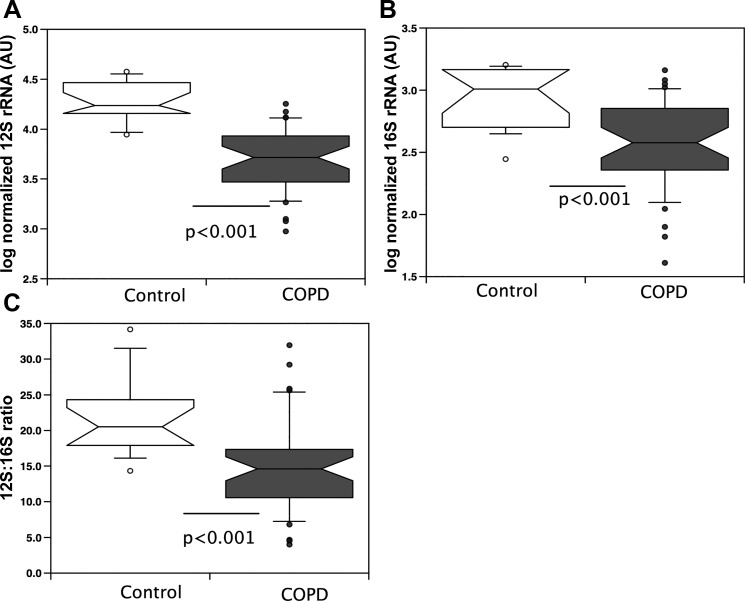
Mitochondrial RNA expression is reduced in chronic obstructive pulmonary disease (COPD) patients. Quadriceps expression of 12S and 16S rRNAs were quantified in samples from COPD (*n* = 40) and controls (*n* = 14). *A* and *B*: expression of both the 12S rRNA (*A*) and 16S rRNA (*B*) were reduced in the patients compared with controls. AU, arbitrary units. *C*: the reduction in the 12S rRNA was larger than that in the 16S rRNA leading to a reduction in the 12S:16S ratio (*C*).

In patients with established ICUAW the marked elevation of miR-542-3p was associated with a significant reduction in 12S rRNA levels compared with 16S rRNA levels indicative of mitochondrial ribosomal stress (11). To determine if increased miR-542-3p in the quadriceps of COPD patients was associated with evidence of mitochondrial ribosomal stress in COPD, we quantified the expression of 12S and 16S rRNA in the patients with COPD (*n* = 40) and controls (*n* = 14) for which we had appropriate cDNAs. There was a reduction in both the 12S and 16S rRNAs that was larger in the 12S rRNA resulting in a significant reduction in the 12S:16S ratio (Fig. 4).

### miR-542 Expression Is Associated with Mitochondrial Gene Expression and P53 Activity in Patients

We have previously shown that overexpression of miR-542 leads to a reduction in the 12S:16S ratio in cells in culture and in the muscle of mice (11). This observation suggests that increased miR-542 in the muscle of patients with COPD may contribute to the loss of mitochondrial function in COPD muscle. To determine whether miR-542 was associated with mitochondrial gene expression, the recently published array data (41) from samples included in this study was compared with the miRNA expression reported here. Biweight midcorrelation coefficients were calculated for all detected genes using the WGCNA R package and correlations that reached a statistical significance of 0.02 were used for gene set enrichment analysis. The gene set most negatively associated with miR-542 was mitochondrial genes, and this observation was true whether the analysis was performed using the patients only or the whole cohort (Table 4). The gene sets positively correlated with miR-542 included sets associated with the inflammatory response raising the possibility that some aspect of inflammatory signaling contributes to the expression of miR-542 (Table 4). However, there was no simple association of miR-542 expression with inflammatory cytokines (IL-2, IL-6, IL-8, or TNF-α) or with global DNA bound NF-κB p50 or p65 indicating that these cytokines alone are not sufficient to explain the association of miR-542 with disease severity (data not shown). The positively correlated gene sets also included the gene set for P53 activity. As mitochondrial ribosomal stress has been shown to promote P53 activation this observation is consistent with miR-542-driven mitochondrial ribosomal stress activating P53.

**Table 4.** Gene set enrichment for genes associated with miR-542

Hallmark	NES	NOM *P* Value	FDR *Q* Value
Positive			
Epithelial mesenchymal transition	4.212	0.000	0.000
Interferon gamma response	2.879	0.000	0.000
IL2 STAT5 signaling	2.716	0.000	0.000
Inflammatory response	2.621	0.000	0.000
Apoptosis	2.616	0.000	0.001
Coagulation	2.605	0.000	0.001
Complement	2.421	0.000	0.002
TNFα signaling via NF-κB	2.087	0.006	0.013
UV response Dn	2.054	0.002	0.014
Hypoxia	1.913	0.008	0.022
Apical junction	1.775	0.020	0.042
P53 pathway	1.767	0.028	0.040
KRAS signaling up	1.762	0.015	0.038
Negative			
Oxidative phosphorylation	−5.046	0.000	0.000
Fatty acid metabolism	−3.058	0.000	0.000
Adipogenesis	−2.231	0.000	0.002

NES, normalized enrichment score; NOM, nominal; FDR, false discovery rate.

### Does miR-542-3p Inhibit Protein Synthesis?

**Fig. 5.**
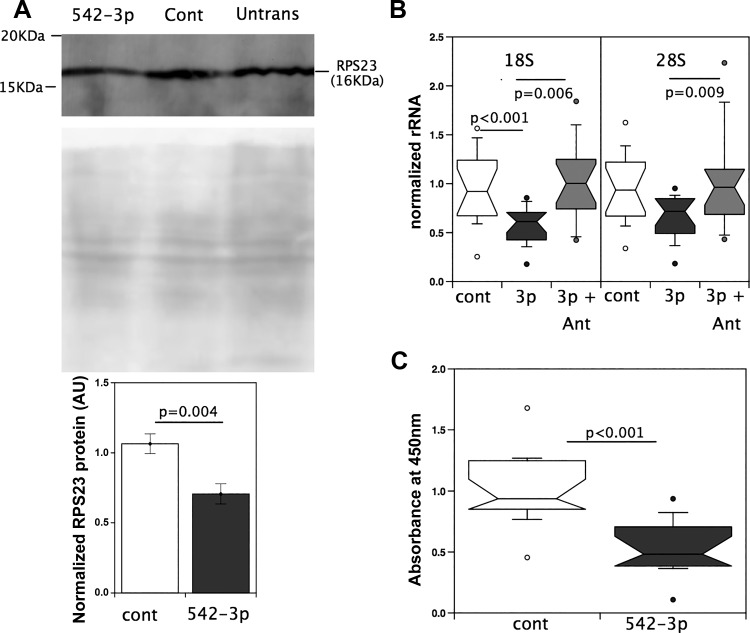
The miR-542-3p suppresses the expression of RPS23 and inhibits protein synthesis in muscle cells. LHCN-M2 cells were transfected with miR-542-3p or scrambled control as described in Methods and incubated for 2 days. *A*: transfected cells were lysed and the expression of RPS23 was determined by Western blotting. The experiment was repeated 3 times and expression of RPS23 was normalized to Ponceau S stain. RPS23 expression was suppressed by transfection with the microRNAs. AU, arbitrary units. *B*: 18S rRNA was quantified and normalized to the geometric mean of β2-microglobiulin and hypoxanthine phosphoribosyltransferase (HPRT) in transfections performed in hextuplicate and the experiment repeated 3 times. miR-542-3p suppressed 18S rRNA expression. *C*: protein synthesis was analyzed by quantifying puromycin incorporation as described in methods with the transfections performed in quadruplicate and the experiment repeated 3 times. Puromycin incorporation was lower in miR-542-3p-transfected cells than in control transfected cells indicating a suppression of protein synthesis. Data are presented as means ± SE. *P* values were calculated by Student’s *t*-test. 3p, miR-542-3p; 3p + Ant, miR-542-3p + antagomiR; cont, control; RPS23, ribosomal protein S23; untrans, untransfected.

miR-542-3p inhibits the expression of a number of proteins that make up the cytoplasmic small ribosomal subunit promoting cytoplasmic ribosomal stress and reducing 18S rRNA expression (39). Consequently, this miRNA should suppress maximal protein synthesis. We therefore confirmed the reduction in RPS23 and 18S rRNA in muscle cells (Fig. 5, *A* and *B*) and determined the effect of miR-524-3p on leucine- and IGF-1-stimulated proteins synthesis by quantifying puromycin incorporation. Transfection of LHCN-M2 muscle myoblasts with miR-542-3p decreased puromycin incorporation (Fig. 5*C*) suggesting that miR-542-3p inhibited the formation of the small ribosomal subunit and inhibited protein synthesis.

## DISCUSSION

The data presented here show that increased expression of miR-542-3p and miR-542-5p is associated with muscle dysfunction in COPD patients. Quadriceps expression of these miRNAs is associated with lung function measured as either TL_CO_% predicted or FEV_1_% predicted. Consistent with promotion of mitochondrial ribosome stress by miR-542-3p, expression of the 12S rRNA was reduced. Furthermore, the expression of miR-542-3p/5p was inversely associated with the expression of mitochondrial genes and positively associated with the genes associated with activation of the p53 pathway, consistent with an association with mitochondrial dysfunction and ribosomal stress. We also show that miR-542-3p can reduce RPS23 and 18S rRNA expression and inhibit protein synthesis in muscle cells. Taken together, the data suggest that disease situated in the lung promotes the muscle expression of miR-542-3p/5p where it promotes mitochondrial dysfunction and inhibits protein synthesis. The association of miR-542-3p/5p with inflammatory signaling raises the possibility that in this context inflammatory mediators released as part of, or in response to, lung disease promote miR-542-3p/5p expression but the lack of correlation of miRNA expression with the classical mediators of inflammatory signaling mean that the factors underlying this association remain to be identified. The promoter that controls the expression of miR-542 has yet to be confirmed but the close proximity of this miRNA to the miR-424-503 host gene suggests that these genes are coexpressed from the same promoter. This suggestion is supported by relatively tight correlation of the expression of miR-542 expression with miR-424 in all the studies we have performed. This region of DNA contains multiple binding sites for STAT, NF-κB, and glucocorticoid receptors suggesting that it functions as part of a stress response system. An alternative possibility for the elevation of these miRNAs in muscle is delivery from another tissue via exosomes. However, this route would require sufficient production of these miRNAs and exosomes in an organ to increase the levels in muscle sixfold and where skeletal muscle accounts for ~40% of the body mass.

### Significance of the Findings

A significant proportion of COPD patients report leg fatigue as a more important locus of exercise limitation than breathlessness (16, 28). A well-recognized cause of this early fatigue is a reduction in mitochondrial function and oxidative capacity. One intriguing component of this reduction in oxidative capacity is a greater reduction in the activity of mitochondrial complex I and IV, which require translation to occur both in the cytoplasm and mitochondria, compared with complex II which only requires translation in the cytoplasm (37). Consequently, a relative reduction in translation in mitochondria would lead to a greater reduction in the activity of complexes I and IV. This reduction in activity of electron transfer components requiring mitochondrial translation is also seen in sarcopenia where an accumulation of mutations in the mitochondrial genome has been suggested to contribute (4, 38). Our previous study showing that miR-542-3p suppressed the expression of mitochondrial ribosomal proteins and the 12S rRNA provided an additional mechanism by which mitochondrial protein synthesis could be suppressed and mitochondrial dysfunction could occur (11). Furthermore, this reduction in the ability to form functional mitochondrial ribosomes also reduced the levels of the small ribosomal RNA (12S) compared with the large ribosomal subunit RNA (16S). Our observation of a reduced 12S:16S rRNA ratio is consistent with such a mechanism contributing to the mitochondrial dysfunction in COPD patients. This effect of miR-542-3p on mitochondrial function is sufficient to explain the association of miR-542-3p with physical performance that we see both in the COPD patients and in the healthy older individuals from the HSS.

This mechanism does not appear to be restricted to COPD patients as we have also demonstrated similar effects in patients with ICUAW (11), in whom there is also preferential loss of complex I and complex IV activity. Taken together, these data suggest that changes mediated by miR-542-3p are not disease specific but a more general pathophysiologic process. In its most extreme form, the effects of inhibition of mitochondrial translation can be seen via the occasional gene mutations in humans causing an inability to synthesize the small subunit of the mitochondrial ribosome (21, 32). These mutations have a similar, but more marked, effect on the electron transfer chain and on 12S rRNA levels to that observed here and ICUAW.

The observation that miR-542-3p-mediated muscle dysfunction can complicate ICUAW, COPD, and indeed, “normal” aging suggests a currently unknown mechanism by which diverse etiologies can trigger a single underlying molecular mechanism. Immobility (or more specifically contractile history) would be one candidate since it is present to some extent in all three conditions. However, we did not see any association of miR-542-3p expression with daily physical activity. Other possible candidates might include inflammation that is present in the intensive care unit and at least episodically in COPD (at time of acute exacerbation), and as demonstrated in this study, there is an association of miR-542 expression with markers of the activation of inflammatory signaling in the muscle but no association with classic inflammatory cytokines. Identification of the mechanisms leading to increased expression of miR-542-3p would be insightful and could offer a novel therapeutic approach to this condition.

### Critique of the Method

Even though we showed that miR-542-3p can suppress maximal protein synthesis in vitro, expression of this miRNA was not strongly associated with FFMI. There are a number of likely reasons for this lack of association. First, FFM is not a good tool for detecting quadriceps weakness in COPD (22), presumably because the muscle wasting observed in COPD is regional in nature. Second, there are multiple inputs into the regulation of protein synthesis and the increase of miR-542-3p would have to be one of the predominant regulators for a significant association to be observed. For example, we have already shown that an miRNA from the same locus as miR-542 (miR-424-5p) suppresses the expression of rRNAs by targeting PolR1A the RNA polymerase required for rRNA synthesis and UBTF, a key transcription factor that activates RNA polymerase I (5) as well as targeting IGF-1 (6). This miRNA while closely correlated with miR-542-3p expression in COPD and ICUAW is associated with FFMI in the COPD patients suggesting that suppression of rRNA synthesis is more important in terms of protein synthesis than the effects of miR-542 on small ribosomal protein expression. Third, altering the number or activity of ribosomes by one miRNA is also unlikely to be the only mechanism contributing to protein synthesis. For example, increasing or reducing the number of nuclei will affect the capacity to transcribe all genes implicating the rate of regeneration in muscle retention. Again, regeneration is controlled by miRNAs, and we have shown that the expression of the imprinted miRNAs from a cluster on chromosome 19 (C19MC; expressed from the paternal chromosome) and miR-675 (expressed from the maternal chromosome) associate with FFMI in COPD patients but not controls (17). Finally, the loss of muscle mass over time is not only due to reduced protein synthesis but protein breakdown also contributes. The rate of protein breakdown will be dependent on the strength of any signal promoting atrophy and the sensitivity of an individual to that signal. Previous studies have shown that levels of GDF-15, a protein that promotes muscle breakdown and reduces appetite, are associated with muscle mass and function in COPD (26) suggesting that this is one proatrophic signal contributing to muscle breakdown. Variation in the sensitivity to atrophic signals also comes from miRNA dependent regulation of the expression of components of signaling pathways with miR-422a reducing the activity of myostatin/TGF-β signaling by suppressing SMAD4 (27).

The work presented here is a cross-sectional study into the expression of a miRNA and mitochondrial rRNAs in patients with COPD. We therefore cannot prove that the associations or relationships are causal. We are also unable to confirm that there is reduced mitochondrial function in the individual samples that we examined as there is insufficient sample available. However, our in vitro and animal studies presented here and in our previous study (11) are consistent with such a role for the miRNAs and there are a number of studies showing reduced mitochondrial function in patients with COPD (reviewed in Ref. 20). Furthermore, we identify an altered 12S:16S ratio in the COPD patients consistent with mitochondrial dysfunction. The data are also consistent with the miRNA suppressing maximal protein synthetic capacity. This altered miRNA and rRNA expression pattern is also seen in the ICUAW patients we have also studied.

In conclusion, our data identify the targeting of both cytoplasmic and mitochondrial ribosomal proteins by miR-542-3p as contributing to the loss of physical performance in patients with COPD. This reduction in ribosomal proteins reduces the availability of small ribosomal subunits inhibiting protein synthesis in each compartment. The expression of these miRNAs is elevated in response to components of the disease process that are known to cause tissue hypoxia, and in vitro data reported here confirm this as a plausible mechanism.

## GRANTS

This work was supported by Medical Research Council (MRC) COPDMAP Grant G1001362, Rosetrees Trust Grant CM500 and the National Institute of Health Research Respiratory Disease Biomedical Research Unit at the Royal Brompton and Harefield National Health Service Foundation Trust and Imperial College London who in part fund M. I. Polley’s salary and wholly fund R. Farre-Garros. J. Y. Lee was wholly supported by COPDMAP. M. Connolly received a British Heart Foundation-funded studentship (FS/14/71/31038). The Hertfordshire Sarcopenia Study is supported by the MRC, and H. Patel received funding from the British Geriatrics Society.

## DISCLAIMERS

The views expressed in this publication are those of the authors and not necessarily those of the National Health Service, National Institute for Health Research, or Department of Health.

## DISCLOSURES

No conflicts of interest, financial or otherwise, are declared by the authors.

## AUTHOR CONTRIBUTIONS

P.R.K. conceived and designed research; R.F.-G., J.Y.L., S.A.N., M.C., and H.P. performed experiments; R.F.-G., J.Y.L., and P.R.K. analyzed data; M.C. and P.R.K. interpreted results of experiments; P.R.K. prepared figures; P.R.K. drafted manuscript; R.F.-G., J.Y.L., S.A.N., M.C., A.A.S., H.P., C.C., M.I.P., and P.R.K. edited and revised manuscript; R.F.-G., J.Y.L., S.A.N., M.C., A.A.S., H.P., C.C., M.I.P., and P.R.K. approved final version of manuscript.

## References

[1] ATS Committee on Proficiency Standards for Clinical Pulmonary Function Laboratories ATS statement: guidelines for the six-minute walk test. Am J Respir Crit Care Med 166: 111–117, 2002. doi:10.1164/ajrccm.166.1.at1102. 12091180

[2] Bergström J Percutaneous needle biopsy of skeletal muscle in physiological and clinical research. Scand J Clin Lab Invest 35: 609–616, 1975. doi:10.3109/00365517509095787. 1108172

[3] Brealey D, Brand M, Hargreaves I, Heales S, Land J, Smolenski R, Davies NA, Cooper CE, Singer M Association between mitochondrial dysfunction and severity and outcome of septic shock. Lancet 360: 219–223, 2002. doi:10.1016/S0140-6736(02)09459-X. 12133657

[4] Bua E, Johnson J, Herbst A, Delong B, McKenzie D, Salamat S, Aiken JM Mitochondrial DNA-deletion mutations accumulate intracellularly to detrimental levels in aged human skeletal muscle fibers. Am J Hum Genet 79: 469–480, 2006. doi:10.1086/507132. 16909385 PMC1559550

[5] Connolly M, Farre Garros R, Paul R, Natanek SA, Bloch S, Sayer AA, Patel HP, Cooper C, Griffiths MJ, Polkey MI, Kemp PR miR-424-5p reduces ribosomal RNA and protein synthesis in muscle wasting. J Cachexia Sarcopenia Muscle 9: 400–416 2018. doi:10.1002/JCSM12266. 29215200 PMC5879973

[6] Connolly M, Garfield BE, Crosby A, Morrell NW, Wort SJ, Kemp PR miR-322-5p targets IGF-1 and is suppressed in the heart of rats with pulmonary hypertension. FEBS Open Bio 8: 339–348, 2018. doi:10.1002/2211-5463.12369. 29511611 PMC5832985

[7] Cooper R, Kuh D, Hardy R; Mortality Review Group; FALCon and HALCyon Study Teams Objectively measured physical capability levels and mortality: systematic review and meta-analysis. BMJ 341: c4467, 2010. doi:10.1136/bmj.c4467. 20829298 PMC2938886

[8] Cote CG, Pinto-Plata V, Kasprzyk K, Dordelly LJ, Celli BR The 6-min walk distance, peak oxygen uptake, and mortality in COPD. Chest 132: 1778–1785, 2007. doi:10.1378/chest.07-2050. 17925409

[9] Cruz-Jentoft AJ, Baeyens JP, Bauer JM, Boirie Y, Cederholm T, Landi F, Martin FC, Michel JP, Rolland Y, Schneider SM, Topinková E, Vandewoude M, Zamboni M; European Working Group on Sarcopenia in Older People Sarcopenia: European consensus on definition and diagnosis: Report of the European Working Group on Sarcopenia in Older People. Age Ageing 39: 412–423, 2010. doi:10.1093/ageing/afq034. 20392703 PMC2886201

[10] Ellis PD, Smith CW, Kemp P Regulated tissue-specific alternative splicing of enhanced green fluorescent protein transgenes conferred by alpha-tropomyosin regulatory elements in transgenic mice. J Biol Chem 279: 36660–36669, 2004. doi:10.1074/jbc.M405380200. 15194683

[11] Farre Garros R, Paul R, Connolly M, Lewis A, Garfield BE, Natanek SA, Bloch S, Mouly V, Griffiths MJ, Polkey MI, Kemp PR miR-542 promotes mitochondrial dysfunction and SMAD activity and is raised in intensive care unit–acquired weakness. Am J Respir Crit Care Med 196: 1422–1433, 2017. doi:10.1164/rccm.201701-0101OC. 28809518 PMC5736972

[12] Frontera WR, Hughes VA, Lutz KJ, Evans WJ A cross-sectional study of muscle strength and mass in 45- to 78-yr-old men and women. J Appl Physiol (1985) 71: 644–650, 1991. doi:10.1152/jappl.1991.71.2.644. 1938738

[13] Gosker HR, Hesselink MK, Duimel H, Ward KA, Schols AM Reduced mitochondrial density in the vastus lateralis muscle of patients with COPD. Eur Respir J 30: 73–79, 2007. doi:10.1183/09031936.00146906. 17428811

[14] Jiroutková K, Krajčová A, Ziak J, Fric M, Waldauf P, Džupa V, Gojda J, Němcova-Fürstová V, Kovář J, Elkalaf M, Trnka J, Duška F Mitochondrial function in skeletal muscle of patients with protracted critical illness and ICU-acquired weakness. Crit Care 19: 448, 2015. doi:10.1186/s13054-015-1160-x. 26699134 PMC4699339

[15] Kemp PR, Griffiths M, Polkey MI Muscle wasting in the presence of disease, why is it so variable? Biol Rev Camb Philos Soc 94: 1038–1055, 2019. doi:10.1111/brv.12489. 30588725

[16] Killian KJ, Leblanc P, Martin DH, Summers E, Jones NL, Campbell EJ Exercise capacity and ventilatory, circulatory, and symptom limitation in patients with chronic airflow limitation. Am Rev Respir Dis 146: 935–940, 1992. doi:10.1164/ajrccm/146.4.935. 1416421

[17] Lewis A, Lee JY, Donaldson AV, Natanek SA, Vaidyanathan S, Man WD, Hopkinson NS, Sayer AA, Patel HP, Cooper C, Syddall H, Polkey MI, Kemp PR Increased expression of H19/miR-675 is associated with a low fat-free mass index in patients with COPD. J Cachexia Sarcopenia Muscle 7: 330–344, 2016. doi:10.1002/jcsm.12078. 27239417 PMC4863928

[18] Lewis A, Riddoch-Contreras J, Natanek SA, Donaldson A, Man WD, Moxham J, Hopkinson NS, Polkey MI, Kemp PR Downregulation of the serum response factor/miR-1 axis in the quadriceps of patients with COPD. Thorax 67: 26–34, 2012. doi:10.1136/thoraxjnl-2011-200309. 21998125 PMC3240776

[19] Man WD, Kemp P, Moxham J, Polkey MI Skeletal muscle dysfunction in COPD: clinical and laboratory observations. Clin Sci (Lond) 117: 251–264, 2009. doi:10.1042/CS20080659. 19681758

[20] Meyer A, Zoll J, Charles AL, Charloux A, de Blay F, Diemunsch P, Sibilia J, Piquard F, Geny B Skeletal muscle mitochondrial dysfunction during chronic obstructive pulmonary disease: central actor and therapeutic target. Exp Physiol 98: 1063–1078, 2013. doi:10.1113/expphysiol.2012.069468. 23377494

[21] Miller C, Saada A, Shaul N, Shabtai N, Ben-Shalom E, Shaag A, Hershkovitz E, Elpeleg O Defective mitochondrial translation caused by a ribosomal protein (MRPS16) mutation. Ann Neurol 56: 734–738, 2004. doi:10.1002/ana.20282. 15505824

[22] Mohan D, Forman JR, Allinder M, McEniery CM, Bolton CE, Cockcroft JR, MacNee W, Fuld J, Marchong M, Gale NS, Fisk M, Nagarajan S, Cheriyan J, Lomas DA, Calverley PMA, Miller BE, Tal-Singer R, Wilkinson IB, Polkey MI; ERICA Consortium Fibrinogen does not relate to cardiovascular or muscle manifestations in COPD: cross-sectional data from the ERICA study. Thorax 73: 1182–1185, 2018. doi:10.1136/thoraxjnl-2018-211556. 29618495

[23] Natanek SA, Gosker HR, Slot IG, Marsh GS, Hopkinson NS, Man WD, Tal-Singer R, Moxham J, Kemp PR, Schols AM, Polkey MI Heterogeneity of quadriceps muscle phenotype in chronic obstructive pulmonary disease (COPD); implications for stratified medicine? Muscle Nerve 48: 488–497, 2013. doi:10.1002/mus.23784. 23553751

[24] Patel H, Syddall HE, Martin HJ, Cooper C, Stewart C, Sayer AA The feasibility and acceptability of muscle biopsy in epidemiological studies: findings from the Hertfordshire Sarcopenia Study (HSS). J Nutr Health Aging 15: 10–15, 2011. doi:10.1007/s12603-011-0006-8. 21267515 PMC12879697

[25] Patel HP, Jameson KA, Syddall HE, Martin HJ, Stewart CE, Cooper C, Sayer AA Developmental influences, muscle morphology, and sarcopenia in community-dwelling older men. J Gerontol A Biol Sci Med Sci 67A: 82–87, 2012. doi:10.1093/gerona/glr020. 21357193

[26] Patel MS, Lee JY, Baz M, Wells CE, Bloch SAA, Lewis A, Donaldson AV, Garfield BE, Hopkinson NS, Natanek SA, Man WD, Wells DJ, Baker EH, Polkey MI, Kemp PR Growth differentiation factor-15 is associated with muscle mass in COPD and promotes muscle wasting in vivo. J Cachexia Sarcopenia Muscle 7: 436–448, 2016. doi:10.1002/jcsm.12096. 27239406 PMC4864181

[27] Paul R, Lee J, Donaldson AV, Connolly M, Sharif M, Natanek SA, Rosendahl U, Polkey MI, Griffiths M, Kemp PR miR-422a suppresses SMAD4 protein expression and promotes resistance to muscle loss. J Cachexia Sarcopenia Muscle 9: 119–128, 2018. doi:10.1002/jcsm.12236. 28984049 PMC5803610

[28] Pepin V, Saey D, Whittom F, LeBlanc P, Maltais F Walking versus cycling: sensitivity to bronchodilation in chronic obstructive pulmonary disease. Am J Respir Crit Care Med 172: 1517–1522, 2005. doi:10.1164/rccm.200507-1037OC. 16166613

[29] Puente-Maestu L, Pérez-Parra J, Godoy R, Moreno N, Tejedor A, González-Aragoneses F, Bravo JL, Alvarez FV, Camaño S, Agustí A Abnormal mitochondrial function in locomotor and respiratory muscles of COPD patients. Eur Respir J 33: 1045–1052, 2009. doi:10.1183/09031936.00112408. 19129279

[30] Rabe KF, Hurd S, Anzueto A, Barnes PJ, Buist SA, Calverley P, Fukuchi Y, Jenkins C, Rodriguez-Roisin R, van Weel C, Zielinski J; Global Initiative for Chronic Obstructive Lung Disease Global strategy for the diagnosis, management, and prevention of chronic obstructive pulmonary disease: GOLD executive summary. Am J Respir Crit Care Med 176: 532–555, 2007. doi:10.1164/rccm.200703-456SO. 17507545

[31] Rabinovich RA, Bastos R, Ardite E, Llinàs L, Orozco-Levi M, Gea J, Vilaró J, Barberà JA, Rodríguez-Roisin R, Fernández-Checa JC, Roca J Mitochondrial dysfunction in COPD patients with low body mass index. Eur Respir J 29: 643–650, 2007. doi:10.1183/09031936.00086306. 17182653

[32] Saada A, Shaag A, Arnon S, Dolfin T, Miller C, Fuchs-Telem D, Lombes A, Elpeleg O Antenatal mitochondrial disease caused by mitochondrial ribosomal protein (MRPS22) mutation. J Med Genet 44: 784–786, 2007. doi:10.1136/jmg.2007.053116. 17873122 PMC2652816

[33] Shrikrishna D, Patel M, Tanner RJ, Seymour JM, Connolly BA, Puthucheary ZA, Walsh SL, Bloch SA, Sidhu PS, Hart N, Kemp PR, Moxham J, Polkey MI, Hopkinson NS Quadriceps wasting and physical inactivity in patients with COPD. Eur Respir J 40: 1115–1122, 2012. doi:10.1183/09031936.00170111. 22362854

[34] Steiner MC, Barton RL, Singh SJ, Morgan MD Bedside methods versus dual energy X-ray absorptiometry for body composition measurement in COPD. Eur Respir J 19: 626–631, 2002. doi:10.1183/09031936.02.00279602. 11998990

[35] Swallow EB, Reyes D, Hopkinson NS, Man WD, Porcher R, Cetti EJ, Moore AJ, Moxham J, Polkey MI Quadriceps strength predicts mortality in patients with moderate to severe chronic obstructive pulmonary disease. Thorax 62: 115–120, 2007. doi:10.1136/thx.2006.062026. 17090575 PMC2111256

[36] Szulc P, Munoz F, Marchand F, Chapurlat R, Delmas PD Rapid loss of appendicular skeletal muscle mass is associated with higher all-cause mortality in older men: the prospective MINOS study. Am J Clin Nutr 91: 1227–1236, 2010. doi:10.3945/ajcn.2009.28256. 20237137

[37] van den Borst B, Slot IG, Hellwig VA, Vosse BA, Kelders MC, Barreiro E, Schols AM, Gosker HR Loss of quadriceps muscle oxidative phenotype and decreased endurance in patients with mild-to-moderate COPD. J Appl Physiol (1985) 114: 1319–1328, 2013. doi:10.1152/japplphysiol.00508.2012. 22815389

[38] Wanagat J, Cao Z, Pathare P, Aiken JM Mitochondrial DNA deletion mutations colocalize with segmental electron transport system abnormalities, muscle fiber atrophy, fiber splitting, and oxidative damage in sarcopenia. FASEB J 15: 322–332, 2001. doi:10.1096/fj.00-0320com. 11156948

[39] Wang Y, Huang JW, Castella M, Huntsman DG, Taniguchi T p53 is positively regulated by miR-542-3p. Cancer Res 74: 3218–3227, 2014. doi:10.1158/0008-5472.CAN-13-1706. 24762395 PMC4058365

[40] Wanger J, Clausen JL, Coates A, Pedersen OF, Brusasco V, Burgos F, Casaburi R, Crapo R, Enright P, van der Grinten CP, Gustafsson P, Hankinson J, Jensen R, Johnson D, Macintyre N, McKay R, Miller MR, Navajas D, Pellegrino R, Viegi G Standardisation of the measurement of lung volumes. Eur Respir J 26: 511–522, 2005. doi:10.1183/09031936.05.00035005. 16135736

[41] Willis-Owen SA, Thompson A, Kemp PR, Polkey MI, Cookson WO, Moffatt MF, Natanek SA COPD is accompanied by co-ordinated transcriptional perturbation in the quadriceps affecting the mitochondria and extracellular matrix. Sci Rep 8: 12165, 2018. doi:10.1038/s41598-018-29789-6. 30111857 PMC6093887

[42] Zhu CH, Mouly V, Cooper RN, Mamchaoui K, Bigot A, Shay JW, Di Santo JP, Butler-Browne GS, Wright WE Cellular senescence in human myoblasts is overcome by human telomerase reverse transcriptase and cyclin-dependent kinase 4: consequences in aging muscle and therapeutic strategies for muscular dystrophies. Aging Cell 6: 515–523, 2007. doi:10.1111/j.1474-9726.2007.00306.x. 17559502

